# Cancer Chemoprevention by Caroteno

**DOI:** 10.3390/molecules17033202

**Published:** 2012-03-14

**Authors:** Takuji Tanaka, Masahito Shnimizu, Hisataka Moriwaki

**Affiliations:** 1 Tohkai Cytopathology Institute, Cancer Research and Prevention (TCI-CaRP), 5-1-2 Minami-Uzura, Gifu 500-8285, Japan; 2 Department of Tumor Pathology, Gifu University Graduate School of Medicine, 1-1 Yanagido, Gifu 501-1194, Japan; 3 Department of Medicine, Gifu University Graduate School of Medicine, 1-1 Yanagido, Gifu 501-1194, Japan

**Keywords:** carotenoids, xanthophylls, cancer chemoprevention, mechanisms

## Abstract

Carotenoids are natural fat-soluble pigments that provide bright coloration to plants and animals. Dietary intake of carotenoids is inversely associated with the risk of a variety of cancers in different tissues. Preclinical studies have shown that some carotenoids have potent antitumor effects both *in vitro* and *in vivo*, suggesting potential preventive and/or therapeutic roles for the compounds. Since chemoprevention is one of the most important strategies in the control of cancer development, molecular mechanism-based cancer chemoprevention using carotenoids seems to be an attractive approach. Various carotenoids, such as β-carotene, α-carotene, lycopene, lutein, zeaxanthin, β-cryptoxanthin, fucoxanthin, canthaxanthin and astaxanthin, have been proven to have anti-carcinogenic activity in several tissues, although high doses of β-carotene failed to exhibit chemopreventive activity in clinical trials. In this review, cancer prevention using carotenoids are reviewed and the possible mechanisms of action are described.

## Abbreviations

ABCA1: ATP-binding cassette transporter 1
AFB_1_: aflatoxin B_1_
Akt: protein kinase B
AMD: age-related macular degeneration
AOM: azoxymethane
AP-1: activator 1
ARE: antioxidant response element
CAR: constitutive androstane receptor
Cdks: cyclin-dependent kinases
CHRP: β-cryptoxanthin- and hesperidin-rich powder
CMO-1: β-carotene 15,15'-monooxygenase
COM2: β-carotene 9',10'-monooxygenase
COX: cyclooxygenase
CUSM: citrus unshiu segment membrane
CVD: cardiovascular disease
CYP: cytochrome P450
DMH: 1,2-dimethylhydrazine
EGF: early growth response gene
ERK: extracellular signal-regulated kinase
GJIC: gap junctional intercellular communication
GSK3β: glycogen synthase kinase 3β
GSTs: glutathione *S*-transferases
HDL: high-density lipoproteins
HO-1: heme oxygenase-1
IGF: insulin growth factor
IGFBPs: IGF binding proteins
IL: interleukin
LDL: low-density lipoproteins
MJ: satsuma mandarin (*Citrus unshiu* Marc) juice
MMP: matrix metalloproteinases
NF-kB: nuclear factor kappaB
4-NQO: 4-nitroquinoline 1-oxide
NQO1: NAD(P)H:quinone oxidoreductase
Nrf2: NF-E2-related factor 2
OH-BBN: *N*-butyl-*N*(4-hydroxybutyl)nitrosamine
PPARs: peroxisome proliferator-activated receptors
PSA: prostate-specific antigen
RAR: retinoic acid receptor
ROS: reactive oxygen species
RXR: retinoid X receptor
SXR/PXR: steroid and xenobiotic receptor/pregnane X receptor
TCF/LEF: transcription factors T cell factor/lymphoid enhancer factor
TNF: tumor necrosis factor
TRE: TPA response element
UV: ultraviolet
VDR: vitamin D3 receptor

## 1. Introduction

To date, the cancer problem and the failure of conventional chemotherapy to achieve a reduction in the mortality rates for common epithelial malignancies such as carcinomas of the lung, colon, breast, prostate and pancreas, indicates a critical need for new approaches to control cancer development [1,2]. One of these approaches is chemoprevention, which is a pharmacological approach to intervention with the objective of arresting or reversing the process of multi-step carcinogenesis. The carcinogenic process may be driven by mutation(s), and followed by subsequent alterations in phenotypic, epigenetic and genetic events. Pharmacologic modulation of these regulatory pathways, involving the effective use of drugs, micronutrients and non-nutrients that block mutational damage of DNA, thus offers great potential for cancer prevention.

**Figure 1:**
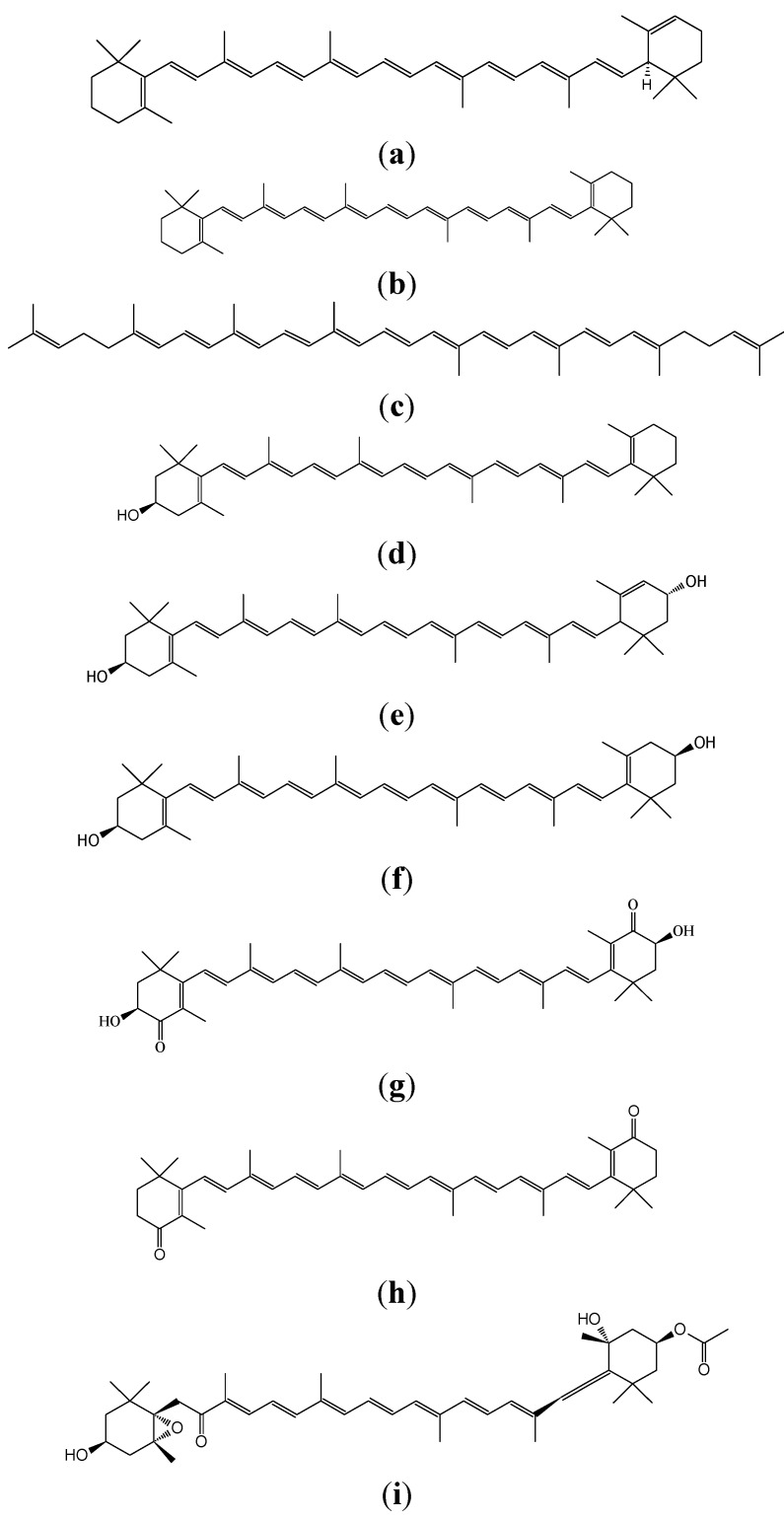
Chemical structures of (**a**) α-carotene; (**b**) β-carotene; (**c**) lycopene; (**d**) β-cryptoxanthin; (**e**) lutein; (**f**) zeaxantin; (**g**) astaxanthin; (**h**) canthaxanthin and (**i**) fucoxanthin.

There is a clear link between dietary intake or dietary habits and cancer development in man [3,4,5]. Dietary risk factors have ranked higher than smoking and much higher than pollution or occupational hazards in their association with death due to cancer [6]. However, a number of compounds naturally occurring in foods, particularly antioxidative compounds in plants, have shown promise as potential chemopreventive agents [2,6,7,8]. These phytonutrients include the yellow, orange and red carotenoid pigments that have recently been investigated. Epidemiologically, vegetable and fruit consumption has constantly been associated with a reduced incidence of a variety of cancers [7,8,9], and dietary carotenoid intake from these sources has similarly been correlated with a reduced cancer risk [10,11,12]. However, several recent large-scale intervention trials failed to find any chemopreventive effects due to long-term supplementation with β-carotene, the most abundant dietary carotenoid [13,14,15]. In contrast, several naturally occurring carotenoids other than β-carotene have exhibited chemopreventive and/or anti-cancer activities [16,17,18,19]. Foodstuffs contain various carotenoids. Vegetables contain carotenoids such as α-carotene (Figure 1a), β-carotene (Figure 1b), lycopene (Figure 1c), β-cryptoxanthin (Figure 1d), lutein (Figure 1e), zeaxanthin (Figure 1f), capsanthin and crocetin. Citrus fruits contain β-cryptoxanthin and marine carotenoids include astaxanthin (Figure 1g), β-carotene, zeaxanthin, canthaxanthin (Figure 1h), fucoxanthin (Figure 1i) and lycopene.

In this brief review, cancer prevention by means of carotenoids (Table 1), are summarized and the possible mechanisms of action are also described.

**Table 1:** Sources, function, and effects of different carotenoids.

Carotenoids	Dietary Sources	Function	Effects
α-Carotene	Yellow-orange vegetables (carrots, sweet totatoes, pumpkin) and Dark-green vegetables (broccoli, green beans, spinach)	Provitamin A activity; Anti-oxidant	Immune- enhancement; Stimulate cell to cell communication; Decreases risk of some cancers
β-Carotene	Green leafy vegetables and orange and yellow fruits and vegetables (carrots, apricots, spinach, sweet potetoes, pumpkin, pepper, kale, cantaloupe)	Provitamin A activity; Antioxidant	Immune-enhancement; Decreases risk of some cancers and some cardiovascular events; high-dose supplementation may increase the risk of lung cancer among smokers
Lycopene	Tomatoes, water melon, apricot, peaches	Anti-oxidant	Decreases risk of some cancers and some cardiovascular events, diabetes, and osteoporosis
β-Cyptoxanthin	Orange fruits (mandarin orange and papaya, *etc*.), corn, peas, and egg yolks	Provitamin A activity; Anti-oxidant	Anti-inflammatory effects; Inhibits risks of some cancer and cardiovascular events; Immune enhancement
Lutein/Zeaxanthin	Dark green leafy vegetables (spinach, kale), red peppers, maize, tomatoes, corn, and egg yolks	Anti-photosensitizing agent and photosynthetic pigment; Acts as antioxidants and blue light filters	Decrease age-related macular degeneration, cataract, and risk of cardiovascular disease and certain cancers
Astaxanthin	Green algae, salmon, trout, crustacea	Antioxidant; Coloration	Prevent certain cancers, cataract, diabetes, and inflammatory neurodegenerative and cardiovascular diseases
Canthaxanthin	Salmon, crustacea	Antioxidant; Coloration	Immune enhancement; Decreases risk of some cancers
Focoxanthin	Brown algae, heterokonts	Antioxidant	Anti-cancer, anti-allergic, anti-obese, anti-inflammatory, and anti-osteoporotic activities

## 2. Definition of Carotenoids

Carotenoids, which belong to the chemical group known as isoprenoid polyenes, are lipid-soluble, yellow-orange-red pigments found in all higher plants and some animals. The carotenoids can be categorized as follows: (a) vitamin A precursors that do not pigment such as β-carotene; (b) pigments with partial vitamin A activity such as cryptoxanthin, β-apo-8'-carotenoic acid ethyl ester; (c) non-vitamin A precursors that do not pigment or pigment poorly such as violaxanthin and neoxanthin; and (d) non-vitamin A precursors that pigment such as lutein, zeaxanthin and canthaxanthin. Due to the numerous conjugated double bonds and cyclic end groups, carotenoids present a variety of stereoisomers with different chemical and physical properties. The most important forms commonly found among carotenoids are geometric (*E*-/*Z*-). A double bond links the two residual parts of the molecule either in an *E*-configuration with both parts on opposite sites of the plane, or a *Z*-configuration with both parts on the same side of the plane. Geometrical isomers of this type are interconvertible in solution. This stereoisomerism exerts a marked influence on the physical properties. Isomers differ not only in their melting points, solubility and stability, but also in respect to absorption affinity, color and color intensity. Animals cannot synthesize carotenoids, so their presence in the body is due to dietary intake of foods such as pink salmon flesh. The plumage of many birds owes its color to carotenoids. Plant, algae, fungal and synthetic (nature-identical) carotenoids are permitted as colorants in food products, but not animal carotenoids.

Carotenoids owe their name to carrots (*Daucus carota*), and xanthophylls (originally phylloxanthins) are derived from the Greek words for yellow (*xanthos*) and leaf (*phyllon*). Together with anthocyanins, carotenoids are the most complex class of natural food colorants with over 750 different structures identified. 

## 3. Absorption, Metabolism, and Bioavailability of Carotenes and Xanthophylls

Carotenoids, being mostly fat soluble, follow the same intestinal absorption path as dietary fat. Carotenoids are released from food matrices and solubilized in the gut. This is carried out in the presence of fat and conjugated bile acids. For carotenoid absorption, as little as 3~5 g of fat in a meal is sufficient [20,21]. Absorption is affected by the same factors that influence fat absorption. Thus, the absence of bile or any generalized malfunction of the lipid absorption system, such as diseases of the small intestine and pancreas, will interfere with the absorption of carotenoids. Chylomicrons are responsible for the transport of carotenoids from the intestinal mucosa to the bloodstream via the lymphatics for delivery to tissues. Carotenoids are transported in the plasma exclusively by lipoproteins. Oxygen functionalized carotenoids are more polar than carotenes. Thus, α-carotene, β-carotene and lycopene tend to predominate in low-density lipoproteins (LDL) in the circulation, whereas high-density lipoproteins (HDL) are major transporters of xanthophylls such as cryptoxanthins, lutein and zeaxanthin [22,23]. The delivery of carotenoids to extrahepatic tissues is accomplished through the interaction of lipoprotein particles with receptors and the degradation by lipoprotein lipase. 

Although no less than forty carotenoids are usually ingested in the diet, only six carotenoids and their metabolites have been found in human tissues, suggesting selectivity in the intestinal absorption of carotenoids [24,25]. In contrast, thirty-four carotenoids and eight metabolites are detected in breast milk and serum of lactating mothers [26]. Recently, facilitated diffusion in addition to simple diffusion has been reported to mediate the intestinal absorption of carotenoids in mammals. The selective absorption of carotenoids may be due to uptake to the intestinal epithelia by means of facilitated diffusion and an unknown mechanism of excretion into the intestinal lumen. It is well known that β-carotene can be metabolized to vitamin A after intestinal absorption of carotenoids, but little is known about the metabolic transformation of non-provitamin A xanthophylls. The enzymatic oxidation of the secondary hydroxyl group leading to keto-carotenoids would occur as a common pathway of xanthophyll metabolism in mammals [24].

## 4. Distribution and Nature of Certain Carotenoids

Numerous studies have reported that carotenoids have the potential to prevent cancers, diabetes, and inflammatory and cardiovascular disease (CVD). Some of these carotenoids are listed below.

### 4.1. Hydrocarbone Carotenoids

Under EU legislation, plant carotenoids may be derived from edible plants, carrots, vegetable oils, grass, alfalfa and nettle. However, according to U.S. legislation carotenes may only be derived from carrots. A good source of plant carotenoids is the mesocarp of oil palm (*Elaeis guineensis*) fruits, which contains an oil rich in carotenes. After separation of the carotenes from the palm fruit oil, which is used for making detergents, the carotenes are suspended in vegetable oil at a concentration of 30%. The predominant carotenes are α- and β-carotene in the ratio 2:3. Other carotenes, including phytoene, phytofluene, ζ-carotene, γ-carotene and lycopene, which are all precursors in the biosynthesis of α- and β-carotene, are present in smaller amounts. Due to heat treatment of the oil palm fruit used in obtaining the oil, a complex mixture of geometric isomers is formed, with only 60% of α- and β-carotene as the *trans*-forms. Synthetic β-carotene is predominantly *trans*-β-carotene. The presence of β-carotene and *cis*-isomers of α- and β-carotene in palm fruit carotenes means that synthetic β-carotene is more orange than palm fruit carotenes, which is more yellow. Carotene from *B. trispora *is also mainly *trans*-β-carotene, with approximately 3% of other carotenoids. Carotene from *D. salina *also primarily consists of β-carotene with 5–6% of other carotenoids (α-carotene, lutein, zeaxanthin and β-cryptoxanthin); according to legislation, the content of *trans*isomers coming from this source should be in the range 50–71%. This means that its color shade would be between that of oil palm carotenes and synthetic β-carotene. Besides being used as colorants, carotenes are also used for nutritional purposes, such as provitamin A agents or as dietary supplements.

β-Carotene is the major source of vitamin A as a provitamin A carotenoid. Two metabolic pathways exist for its conversion to vitamin A, and they are known as the central cleavage pathway and the excentric cleavage pathway. For provitamin A carotenoids, central cleavage is the main pathway leading to the formation of vitamin A [27,28]. β-Carotene, α-carotene, and β-cryptoxanthin are cleaved symmetrically at their central double bond by β-carotene 15,15'-monooxygenase (CMO1), formerly called β-carotene 15,15'-dioxygenase. An alternative excentric cleavage pathway was also reported [29,30] and confirmed by molecular identification of an excentric cleavage enzyme, β-carotene 9',10'-monooxygenase (CMO2) in mice, humans, and zebrafish [31]. CMO2 has the ability to catalyze the asymmetric cleavage of β-carotene to produce β-apo-10'-carotenal and β-ionone [31]. Apo-β-carotenals can be precursors of vitamin A *in vitro* and *in vivo*, by further cleavage enzyme, CMO1 [32]. They can also be oxidized to their corresponding apo-β-carotenoic acids, which undergo a process similar to β-oxidation of fatty acids, to produce retinoic acid [33]. The coexistence of these two cleavage pathways reveals a greater complexity of β-carotene metabolism in organisms and raises a potential link between effects from β-carotene and/or its metabolites and anti-carcinogenesis. Common non-synonymous single-nucleotide polymorphisms (SNPs) exist in the human CMO1 gene and alter β-carotene metabolism [34,35].

### 4.2. Lycopene

Being a precursor in the biosynthesis of β-carotene, lycopene can be expected to be found in plants containing β-carotene, albeit usually at very low and sometimes undetectable concentrations. Thebest-known sources of lycopene are tomatoes, watermelon, guava and pink grapefruit. Lycopene mayalso be produced synthetically and by *B. trispora*. Lycopene is permitted as a food colorant in the EU and was also approved for use as a food supplement in the USA in July 2005. The only permitted source is tomatoes (*Lycopersicon esculentum*, Lycopersicon, meaning wolf peach). Besides lycopene, tomato oleoresin also contains appreciable amounts of β-carotene, phytoene and phytofluene. In solution, lycopene appears orange and not bright red as in the tomato. Lycopene is very prone to oxidative degradation, much more so than β-carotene. 

Carotenoids absorb light, transfer energy to chlorophyll in the process of photosynthesis and protect against photo-oxidative damage [36,37]. In man, carotenoids function primarily as dietary sources of provitamin A. However, lycopene lacks the β-ionone ring structure required to form vitamin A and has no provitamin A activity. Therefore, lycopene has no known physiological function in man. However, some potential molecular targets in cells have been identified for lycopene. They include molecules that are involved in antioxidant activity, the antioxidant response element (ARE), apoptosis induction, cell cycle arrest, growth factors and signaling pathways, and invasion and metastasis [38,39,40,41,42].

### 4.3. Lutein and Zeaxanthin

Lutein and zeaxanthin are the two major components of the macular pigments of the retina. The macula lutea “yellow spot” in the retina is responsible for central vision and visual activity. Lutein and zeaxanthin are the only carotenoids found in both the macula and lens of the human eye, and have dual functions in both tissues to act as powerful antioxidants and to filter high-energy blue light [43]. Lutein is found in high amounts in human serum [26]. In the diet it occurs in highest concentrations in dark green leafy vegetables (spinach, kale, collard greens and others), corn and egg yolks [44]. Zeaxanthin is the major carotenoid found in corn, orange peppers, oranges and tangerines. 

Lutein is also a very common carotenoid and one of the major xanthophylls present in green leafy vegetables. Lutein and zeaxanthin are known to selectively accumulate in the macula of the human retina. They are thought to function as antioxidants [45,46] and as blue light filters [47] to protect the eyes from oxidative stresses such as cigarette smoke and sunlight, which can lead to age-related macular degeneration (AMD) and cataracts. The name lutein is derived from the Latin word for yellow (compare xanthophyll, vide supra). The most interesting source is Aztec marigold (*Tagetes erecta*) in which lutein is primarily found esterified with saturated fatty acids (lauric, myristic, palmitic and stearic acid). Lutein made from Aztec marigold also contains some zeaxanthin (typically less than 10%). Containing only 10 conjugated double bonds, lutein is more yellowish-green than oil palm carotenes. 

Zeaxanthin, the principal pigment of yellow corn, *Zeaxanthin mays *L. (from which its name is derived) is the compound that consists of 40 carbon atoms. It also occurs in egg yolks and some of the orange and yellow vegetables and fruits, such as alfalfa and marigold flowers [48]. Zeaxanthin exhibits no vitamin A activity. Zeaxanthin and its close relative lutein play a critical role in the prevention of AMD, the leading cause of blindness [49]. Zeaxanthin is isomeric with lutein; the two carotenols only differ from each other in terms of the shift of a single double bond, so that in zeaxanthin all double bonds are conjugated. Zeaxanthin is used as a feed additive and colorant in the food industry for birds, swine and fish [50]. The pigment imparts a yellow coloration to the skin of birds and their egg yolk, whereas in pigs and fish it is used for skin pigmentation [51].

### 4.4. β-Cryptoxanthin

β-Cryptoxanthin is found in human blood together with α-carotene, β-carotene, lycopene, lutein and zeaxanthin. Unlike other abundant carotenoids, β-cryptoxanthin is not found in most fruits or vegetables but only in specific ones, namely hot pepper, persimmon and Satsuma mandarin (*Citrus unshiu *Marc.) [52]. Satsuma mandarin, also known as table orange or Satsuma in Western countries, is one of the most popular citrus fruits in Japan. It is sweet, tasty and rich in vitamin C. It is notable that Satsuma mandarin is one of the most common β-cryptoxanthin rich fruits in the world. The edible part of the Satsuma mandarin contains about 1.8 mg/100 g of β-cryptoxanthin, while the β-cryptoxanthin content is 0.2 mg/100 g in Valencia orange and almost nothing in grapefruits. As β-cryptoxanthin is rarely found in most fruits or vegetables, the serum β-cryptoxanthin concentration in the Japanese population is almost parallel to their consumption of the Satsuma mandarin, and is higher than in western populations [53]. Although the nutritional functions and metabolism of abundant carotenoids, for example β-carotene and lycopene, have been well studied [54,55], those of β-cryptoxanthin have not been examined in detail. Recent reports strongly suggest a significant negative correlation between serum β-cryptoxanthin concentrations and disease morbidity such as liver disorders [56,57], cancer [58,59] and mutagenesis [60], and post-menopausal osteoporosis [61,62,63]. β-Cryptoxanthin intake is beneficial for human health. The anti-obesity effects of β-cryptoxanthin have recently been reported [64,65]. The major xanthophyll, β-cryptoxanthin, was also reported to decrease the gene expression of interleukin (IL)-1α in mouse macrophage RAW264 cells [66], to promote osteoblastic differentiation of mouse MC3T3 cells [67] and to prevent a decrease of calcium content in the bone of ovariectomized rats [63].

### 4.5. Astaxanthin

Astaxanthin contains two keto groups on each ring structure as compared with other carotenoids, resulting in enhanced antioxidant properties. This compound occurs naturally in a wide variety of living organisms including microalgae (*Haematococcus pluvialis*, *Chlorella zofingiensis* and *Chlorococcum* sp.), fungi (*Phaffia rhodozyma*, red yeast), complex plants, seafood and some birds such as flamingos and quail; it has a reddish color and gives salmon, shrimp and lobster their distinctive coloration [68]. The microalga *Haematococcus pluvialis *has the highest capacity to accumulate astaxanthin at up to 4–5% of cell dry weight. Astaxanthin has been attributed with the extraordinary potential of protecting the organism against a wide range of diseases. It also has considerable potential and promising applications in the prevention and treatment of various diseases such as cancers, chronic inflammatory diseases, metabolic syndrome, diabetes, diabetic nephropathy, CVD, gastrointestinal and liver diseases, and neurodegenerative diseases [69]. Astaxanthin cannot be manufactured in animals or converted to vitamin A, and therefore must be consumed in the diet. Astaxanthin and canthaxanthin have antioxidant activity, are free radical scavengers, potent quenchers of reactive oxygen species (ROS) and nitrogen oxygen species, and chain-breaking antioxidants. They are superior antioxidants and scavengers of free radicals as compared with the carotenoids such as β-carotene [70]. Astaxanthin is even called superantioxidant.

### 4.6. Canthaxanthin

Canthaxanthin was first isolated from the edible mushroom, *Cantharellus cinnabarinus*. In addition, canthaxanthin is said to be produced at the end of the growth phase in several green algae, and also in blue-green algae, as secondary carotenoids instead of, or in addition to, primary carotenoids. It has also been found in bacteria, crustacea and various species of fish including carp (*Cyprinus carpio*), golden mullet (*Mugil auratus*), annular seabream (*Diplodus annularis*) and trush wrasse (*Crenilabrus tinca*). Canthaxanthin is not encountered in wild Atlantic salmon, but represents a minor carotenoid in wild Pacific salmon. It has also been reported in wild trout (*Salmo trutta*). Canthaxanthin is used widely as a drug or as a food and cosmetic colorant (skin tanning), but it may have some undesirable effects on human health. These are mainly caused by the formation of crystals in the *macula lutea* membranes of the retina. This condition is called canthaxanthin retinopathy [71]. It has been shown that this type of dysfunction of the eye is strongly connected with damage to the blood vessels around the locations of crystal deposition. 

Canthaxanthin is one of the carotenoids without provitamin A activity, but may have anti-carcinogenic, immune-enhancing, antioxidative activities. The mechanisms by which canthaxanthin may exert anti-tumor activity are associated with its antioxidant properties through radical trapping or chain-breaking processes [72,73], or its enhancement of gap-junction cell to cell communication through upregulation of the gap-junction protein, connexin [74]. 

### 4.7. Fucoxanthin

The allenic carotenoid fucoxanthin is one of the most abundant carotenoids, and contributes to nature more than 10% of the estimated total production of carotenoids in nature, especially in the marine environment [75]. Fucoxanthin is a naturally occurring brown- or orange-colored pigment that belongs to the class of non-provitamin A carotenoids. Fucoxanthin acts as an antioxidant under anoxic conditions. The typical antioxidants are usually proton donors (ascorbic acid, α-tocopherol and glutathione). Fucoxanthin, on the other hand, donates an electron as a part of its free-radical quenching function. A combination of these distinct properties is very rarely found among naturally occurring compounds [76,77]. During normal metabolism the body produces heat. Fucoxanthin increases the amount of energy released as heat in fat tissue, a process known as thermogenesis. In a published study it has been reported that fucoxanthin affects multiple enzymes involved in fat metabolism causing an increase in the production of energy from fat [78].

Fucoxanthin is present in *Chromophyta *(*Heterokontophyta *or *Ochrophyta*), including brown seaweeds (*Phaeophyceae*) and diatoms (*Bacillariophyta*) [79]. Based on its unique molecular structure, fucoxanthin has remarkable biological properties similar to neoxanthin, dinoxanthin and peridinin, which make it different to other carotenoids. Fucoxanthin does not exhibit toxicity and mutagenicity under experimental conditions [79,80,81]. Fucoxanthin may have the ability to increase circulating cholesterol levels in rodents as a common feature [79].

## 5. Clinical Trials with Long-Term β-Carotene Supplementation

Epidemiologic studies have shown an inverse relationship between the presence of various cancers and dietary or blood carotenoid levels [82]. However, three [13,14,15] out of four intervention trials [13,14,15,83] using high-doses of β-carotene supplements did not show protective effects against cancer or CVD. Rather, the high-dose intervention trials showed an increase in cancer and angina pectoris [13,14,15,83]. Therefore, carotenoids may promote health when taken at dietary levels, but may have adverse effects when taken high doses by subjects who smoke or who have been exposed to asbestos.

The epidemiologic observations of the possible protective effects of high dietary (not supplemental) β-carotene intakes against cancer, along with what is known about carotenoid biochemical functions, has led to further study of the effect of β-carotene on cancer risk. Long-term large randomized intervention trials were designed to test the efficacy of high doses of β-carotene (20–30 mg/day) in the prevention of cancer (Table 2). As stated above, the results from two trials provided possible evidence of harm from β-carotene supplements in relation to cancer among high-risk individuals such as smokers and asbestos workers [15], but no effect (either beneficial or detrimental) in a generally well-nourished population [84]. Moreover, the Linxian (Chinese) Cancer Prevention Study [83] found that supplementation with β-carotene, vitamin E and selenium led to a significant reduction in total mortality (9%), especially from cancer (13%) and stomach cancer in particular (21%) (Table 2). The positive results of the Chinese study probably reflect the correction of a vitamin A deficiency in the study population. A number of mechanisms have been proposed to account for the association between β-carotene supplementation and lung cancer in smokers and asbestos workers, including an imbalance of other carotenoids or antioxidants, a pro-oxidant activity of β-carotene at the high oxygen tensions found in the lungs, induction of P450 enzymes and the production of damaging β-carotene oxidation products by components of cigarette smoke [85]. The Women’s Health Study [86] indicated no statistically significant differences in incidence of cancer, CVD, or total mortality, although the treatment duration is short (a median treatment duration of 2.1 years and a median total follow-up of 4.1 years).

**Table 2:** β-Carotene supplementation trials.

Studies	Study Designs				Ref. No.
	Population	Intervention	Duration	Cancer outcome	
ATBC	29,133 Finish male smokers (50–69 years of age)	β-carotene, 20 mg/day; vitamin E, 50 mg/day	5–8 years	18% increase in lung cancer; 8% increase in mortality	13
CARET	18,314 men and women and asbestoss workers (45–74 years of age)	β-carotene, 30 mg/day; vitamin A, 25,000 IU	<4 years	28% increase in lung cancer; 17% increase in deaths	15
PHS	22,071 male physicians (40–84 years of age)	β-carotene, 50 mg on alternate days	12 years	No effect of supplementation in incidence of cancer	14
Linxian	29,584 men and women, vitamin and mineral deficient (40–69 years of age)	β-carotene, 15 mg/day; selenium, 50 mg/day; α-tocopherol, 30 mg/day	5 years	13% decrease in total cancers; 9% decrease in overall deaths	84
Women’s Health Study	39,876 female health professionals (over 45 years of age)	β-carotene, 50 mg on alternate days	4.1 years (2.1 years’ treatment and 2.0 years’ follow-up)	No effect of supplementation in incidence of cancer	87

The epidemiologic studies reported an inverse relationship between diet and/or blood β-carotene levels and cancer prevention. It is probable that β-carotene serves as a marker of increased fruit and vegetable intake and, therefore, of all components that have cancer prevention potential, for example vitamin C, folic acid, other carotenoids and polyphenols. Alternatively, low-dose dietary levels could have a protective effect against cancer, whereas high-dose β-carotene supplementation could have a cancer stimulating effect.

## 6. Cancer Chemoprevention by Carotenoids in Preclinical Studies

Cancer chemoprevention is a rapidly expanding discipline that focuses on the discovery and identification of dietary agents and drugs that prevent or inhibit malignant tumor development [4,5]. Since approximately one-third of the overall risk of cancer is attributable to diet, a large number of dietary compounds have been tested to determine their chemopreventive ability using animal carcinogenesis models [87,88,89,90]. The higher eukaryotic aerobic organisms, including man, cannot exist without oxygen, yet oxygen represents a danger to their very existence due to its high reactivity. This fact has been termed the paradox of aerobic life [91]. A number of ROS are generated during normal aerobic metabolism such as the superoxide, hydrogen peroxide and the hydroxyl radical. In addition, singlet oxygen can be generated through photochemical events (in skin and eyes), and lipid peroxidation can lead to peroxyl radical formation [92]. These oxidants collectively contribute to aging and degenerative diseases such as cancer and atherosclerosis through oxidation of DNA, proteins and lipids [91,92,93]. Antioxidant compounds can decrease mutagenesis, and thus carcinogenesis, both by decreasing oxidative damage of DNA and by decreasing oxidant-stimulated cell division [92]. The human body maintains an array of endogenous antioxidants such as catalase and superoxide dismutase; however, exogenous dietary antioxidants such as ascorbic acid (vitamin C), α-tocopherol (vitamin E) and carotenoids play important roles in reducing oxidative damage as well [91,92,93], and their serum levels have the potential to be manipulated [93]. Major carotenoids with antioxidant activity that have been extensively evaluated with regard to their cancer chemopreventive ability include α- and β-carotenes, β-cryptoxanthin, lycopene, lutein and zeaxanthin.

### 6.1. α- and β-Carotene

Carotenoids have been studied vigorously to see if these colorful compounds can decrease the risk of cancer. In ecological studies and early case-control studies it appeared that β-carotene was a cancer-protective agent. Randomized controlled trials of β-carotene found that the isolated nutrient was either without effect [14] or actually increased the risk of lung cancer in smokers [13,15]. β-Carotene may be a marker for the intake of fruits and vegetables, but it does not have a powerful protective effect in isolated pharmacological doses. However, there is a large body of literature indicating that dietary carotenoids are cancer preventative. α-Carotene has been found to be a stronger protective agent than its well-known isomer β-carotene [94]. Studies tend to agree that overall intake of carotenoids is more protective than a high intake of a single carotenoid [94]. Hence, a variety of fruits and vegetables is still a better anti-cancer strategy than just using a single vegetable high in a specific carotenoid [94]. The richest source of α-carotene is carrots and carrot juice, with pumpkins and winter squash as a second densest source [94]. There is approximately 1 μg of α-carotene for every 2 μg of β-carotene in carrots. Previous studies in our laboratory have demonstrated the chemopreventive ability of β-carotene against oral carcinogenesis in rats [95].

Several experimental animal studies have shown that α-carotene possesses higher activity than β-carotene in suppressing tumorigenesis in the skin, lung, liver and colorectum [18,96]. In a skin tumorigenesis experiment conducted by Murakoshi *et al.* [18], the incidence of tumor-bearing mice in the positive control group was 69%, whereas those in the groups treated with β- and α-carotene were 13% and 25%, respectively. The average multiplicity (number of tumors/mouse) of tumors in the positive control group was 3.73/mouse, whereas the α-carotene-treated group had 0.13/mouse (*p* < 0.01). β-Carotene treatment also decreased tumor multiplicity (1.31/mouse), but the difference from the positive control group was insignificant (*p* < 0.05). The higher potency of α-carotene relative to β-carotene in the suppression of tumor promotion was further confirmed in their studies [18]. In a mouse lung carcinogenesis model initiated by 4-nitroquinoline 1-oxide (4-NQO) and promoted by glycerol, the average multiplicity of lung tumors per mouse in the positive control group was 4.06/mouse, whereas the α-carotene-treated group had 1.33/mouse (*p *< 0.001). β-Carotene treatment did not show any suppressive effect on tumor multiplicity, which was significantly increased (4.93/mouse, *p* < 0.02). In their liver carcinogenesis experiment [18], male C3H/He mice, which have a high incidence of spontaneous liver tumor development, were treated with drinking water containing 0.05% α- and β-carotene for 40 weeks. The mean number of hepatomas (3.00/mouse; *p *< 0.001) in the mice that received α-carotene was significantly decreased as compared with the untreated control group (6.31/mouse). On the other hand, the β-carotene-treated group only showed a tendency toward a decrease in tumors (4.71/mouse), as compared with the control group [18]. Narisawa *et al.* [96] also demonstrated the protective effects of α-carotene, lycopene and lutein, but not β-carotene, on preneoplastic colorectal adenocarcinoma lesions. 

### 6.2. β-Cryptoxanthin

It is known that certain carotenoids and flavonoids can inhibit cancer development in animal carcinogenesis models [87,88,89,90]. β-Cryptoxanthin and hesperidin are such compounds. β-Cryptoxanthin with non-substituted β-ionone cycles and provitamin A properties exhibits several biological activities, including the scavenging of free radicals, enhancement of gap junctions, immunomodulation and regulation of the enzyme activity involved in carcinogenesis [97,98]. The most common sources of β-cryptoxanthin are citrus fruits and red sweet peppers. β-Cryptoxanthin is reported to inhibit mouse skin tumorigenesis [58] and rat colon carcinogenesis [99]. Narisawa *et al.* also reported that 25 ppm of β-cryptoxanthin administered for 30 weeks in the diet significantly suppressed *N*-methylnitrosourea-induced colon carcinogenesis in rats [99]. This suggested that dietary β-cryptoxanthin may affect colon carcinogenesis after accumulation in the colonic mucosa, perhaps due to absorption from the colon as well as the small intestine. β-Cryptoxanthin-rich juice (Satsuma mandarin juice [MJ]) has also been found to inhibit colon [59] and lung [100] carcinogenesis. Hesperidin, present in several vegetables and fruits, has antioxidant properties, and anti-inflammatory and inhibitory effects on prostaglandin biosynthesis. This flavonoid has been shown to inhibit chemically induced carcinogenesis in several organs [87,88,89,90,101,102,103,104,105]. β-Cryptoxanthin and hesperidin are thus considered to be potential cancer chemopreventive compounds. However, edible plants contain only small amounts of these chemicals. Therefore, to obtain higher contents of these compounds in foods we prepared a pulp (CHRP) containing high amounts of β-cryptoxanthin and hesperidin during the process of making MJ. CHRP (100 g) contained 0.67 g of β-cryptoxanthin and 3.58 g of hesperidin; the contents of β-cryptoxanthin and hesperidin were 583 times and 38 times greater than those in the edible parts of Satsuma mandarin, respectively. In addition, we prepared Satsuma mandarin juices, which we called MJ2 (1.7 mg of β-cryptoxanthin and 84 mg of hesperidin/100 g) and MJ5 (84 mg of β-cryptoxanthin and 100 mg of hesperidin/100 g), by adding CHRP to the standard Satsuma mandarin juice (MJ: 0.8 mg of β-cryptoxanthin and 79 mg of hesperidin/100 g). We have demonstrated the chemopreventive effects of CHRP and MJs on chemically induced oncogenesis in rat colon and tongue and mouse lung [59,100,106,107]. 

Citrus compounds act on multiple key elements in signal transduction pathways related to cellular proliferation, differentiation, apoptosis, inflammation and obesity. We have found that *Citrus unshiu* segment membrane (CUSM) containing β-cryptoxanthin and fiber suppresses colitis- and obesity-related colon tumorigenesis in animal models [108,109]. Feeding involving a diet with CUSM treatment also decreased the serum level of triglycerides.

### 6.3. Lycopene

There are relatively few reports on the cancer chemopreventive effects of lycopene or other tomato carotenoids in animal models. The majority, but not all, of these studies have indicated a protective effect. Inhibitory effects were seen in two studies using aberrant crypt foci (putative precursors of colon cancer) [96] and colon cancer [110] as biomarkers, and in two mammary tumor studies, one using the dimethylbenz(*a*)anthracene model [111] and the other the spontaneous mouse model [112]. Inhibitory effects were also reported in mouse lung [113] and rat hepatocarcinoma [114] and bladder cancer [115] models. However, a study by Cohen *et al.*[116] found no effect in the *N*-nitrosomethylurea-induced mammary tumor model when crystalline lycopene or a lycopene-rich tomato carotenoid oleoresin was administered in the diet. Unfortunately, differences in routes of administration (gavage, intraperitoneal injection, intra-rectal instillation, drinking water and diet supplementation), species and strain differences, form of lycopene (pure crystalline, beadlet and mixed carotenoid suspension), varying diets (grain-based and casein based) and dose ranges (0.5–500 ppm) resulted in no prevention effect on development of chemically induced mammary cancer. It is clear that the majority of ingested lycopene is excreted in the feces and that 1,000-fold more lycopene is absorbed and stored in the liver than in other target organs. Nonetheless, physiologically significant (nanogram) levels of lycopene are assimilated by key organs such as breast, prostate, lung and colon, and there is a rough dose-response relationship between lycopene intake and blood levels. Pure lycopene was absorbed less efficiently than the lycopene-rich tomato carotenoid oleoresin, and blood levels of lycopene in rats fed a grain based diet were consistently lower than those in rats fed lycopene in a casein-based diet. The latter suggests that the matrix in which lycopene is incorporated is an important determinant of lycopene uptake. 

High intake of lycopene has been associated with a lower risk of a variety of cancers including lung cancer. Lycopene can be converted to apo-10'-lycopenoids [117] in mammalian tissues and can be cleaved by carotene 9',10'-oxygenase at its 9',10' double bond to form apo-10'-lycopenoids, including apo-10'-lycopenal, apo-10'-lycopenol, and apo-10'-lycopenoic acid. Among apo-10'-lycopenoids, apo-10'-lycopenoic acid has been recently shown to inhibit lung carcinogenesis both *in vivo *and *in vitro* [118]. Since enzymatic metabolites of lycopene induce NF-E2-related factor 2 (Nrf2)-mediated expression of phase II detoxifying/antioxidant enzymes including heme oxygenase-1 (HO-1), NQO1, GSTs, and glutamate-cysteine ligases in human bronchial epithelial cells, BEAS-2B [119] and human liver cell cancer cells, HepG2 [120], the anti-carcinogenic and antioxidant functions of lycopene are mediated by apo-10'-lycopenoids, especially apo-10'-lycopenoic acid, via activating Nrf2 and inducing phase II detoxifying/antioxidant enzymes [119]. 

Of the various carotenoids lycopene has been found to be very protective, particularly for prostate cancer. The major dietary source of lycopene is tomatoes, with the lycopene in cooked tomatoes being more bioavailable than that in raw tomatoes. Several prospective cohort studies have found associations between high intake of lycopene and reduced incidence of prostate cancer, although not all studies have produced consistent results [121,122]. Some studies suffer from a lack of good correlation between lycopene intake assessed by questionnaire and actual serum levels, and other studies measured intakes among a population that consumed very few tomato products. In the Health Professionals Follow-up Study there was a 21% decrease in prostate cancer risk, when comparing the highest quintile of lycopene intake with the lowest quintile. Combined intake of tomatoes, tomato sauce, tomato juice and pizza (which accounted for 82% of the lycopene intake) was associated with a 35% lower risk of prostate cancer. Furthermore, lycopene was even more protective for advanced stages of prostate cancer, with a 53% decrease in risk [123]. A more recent follow-up report on this same cohort of men confirmed these original findings that lycopene or frequent tomato intake is associated with about a 30–40% decrease in the risk of developing prostate cancer, especially advanced prostate cancer [124]. In addition to the two reports detailed above, a nested case control study from the Health Professional Follow-up Study involving 450 cases and controls found an inverse relationship between plasma lycopene and prostate cancer risk (OR 0.48) among older subjects (>65 years of age) without a family history of prostate cancer [125]. 

In addition to these observational studies, two clinical trials have been conducted to supplement lycopene for a short period before radical prostatectomy. In one study 30 mg/day of lycopene were given to 15 men in the intervention group, while 11 men in the control group were instructed to follow the National Cancer Institute’s recommendations to consume at least five servings of fruits and vegetables daily. Results showed that lycopene slowed the growth of prostate cancer. Prostate tissue lycopene concentration was 47% higher in the intervention group. Subjects that took lycopene for 3 weeks had smaller tumors, less involvement of the surgical margins and less diffuse involvement of the prostate by pre-cancerous high-grade prostatic intraepithelial neoplasia [126]. In another study carried out before radical prostatectomy surgery, 32 men were given a tomato sauce-based pasta dish every day, which supplied 30 mg of lycopene per day. After 3 weeks serum and prostate lycopene levels had increased 2-fold and 2.9-fold, respectively. Prostate-specific antigen (PSA) levels had decreased by 17%, as also reported by Kucuk *et al*. [126]. Oxidative DNA damage was 21% lower in the patients’ leukocytes and 28% lower in prostate tissue, as compared with the non-study controls. The apoptotic index was 3-fold higher in the resected prostate tissue, relative to biopsy tissue [127]. 

A number of issues remain to be resolved before any definitive conclusions can be drawn concerning the anticancer effects of lycopene. These include the following: the optimal dose and form of lycopene; interactions among lycopene and other carotenoids and fat soluble vitamins such as vitamin E and D; the role of dietary fat in regulating lycopene uptake and disposition; organ and tissue specificity; and the problem of extrapolation from rodent models to human populations [128].

### 6.4. Lutein and Zeaxanthin

In addition to playing pivotal roles in ocular health, lutein and zeaxanthin are important nutrients for the prevention of CVD, stroke and lung cancer. They may also be protective in skin conditions attributed to excessive ultraviolet (UV) light exposure. In a 10-year study following 120,000 U.S. men and women, a significant reduction in lung cancer was observed in patients with the highest intake of total carotenoids including lutein and zeaxanthin [129]. A second 14-year study assessed the same relationship in 27,000 Finnish male smokers via a food-item questionnaire. Consumption of carotenoid containing fruits and vegetables was associated with a decreased risk of lung cancer. A decreased risk of lung cancer was also observed in individuals in the highest quintiles of lutein/zeaxanthin intake *versus* the lowest quintiles. A population-based survey of 20 South Pacific Island populations examined the association between lutein consumption and lung cancer rates. Researchers found an inverse association between lutein and lung cancer and a markedly lower incidence rate for lung cancer among Fijians, as compared with other South Pacific populations. Fijians consume an average of 200 g of dark green vegetables (25 mg lutein) daily; whereas inhabitants of other South Pacific countries consume diets in which colorful fruits and vegetables are less plentiful [130].

Carotenoids singly or in combination could lower cancer risk due to their antimutagenic properties and ability to scavenge free radicals, to protect against tumor development and to improve immune response [131,132]. Lutein and β-carotene quench peroxy radicals and demonstrate antioxidant properties against oxidative damage *in vitro* [133,134]. Plasma lutein analyzed from 37 women correlated inversely with measured oxidative indices [135]. It has been shown *in vitro* using multilamellar liposomes, that carotenoids in combination elicit a greater antioxidant defense than singly. The strongest synergistic effect was obtained in the presence of lutein or lycopene [136]. Lutein may be anticarcinogenic as well. This is suggested by its ability to interact with the mutagens 1-nitropyrene and aflatoxin B_1_ (AFB_1_) [137,138]. Lutein may also exert an anticarcinogenic effect by stimulating certain genes involved in T-cell transformations activated by mitogens, cytokines and antigens [139].

Investigation of lutein’s protective effects in relation to site-specific cancers is beginning to evolve in epidemiologic studies and animal models. No associations have been detected between plasma lutein and zeaxanthin concentrations and gastric cancer [140]. Slattery *et al*. [141] detected an inverse association between dietary lutein intake and colon cancer in men and women. The reduction in risk was significant only in patients who were diagnosed with colon cancer at a younger age [141]. Carotenoid esters are found in human skin [142]. A combination of carotenoids may protect against the development of erythema in human skin [143] and are correlated with the presence or absence of skin cancer and precancerous lesions [141]. The specific effects of lutein on skin cancer are yet to be determined. Previous research has shown modest relationships between the consumption of nutrients found in carotenoid rich foods such as β-carotene and vitamin A, and a reduced risk of breast cancer [144,145,146]. Focus on the potential protective effects of lutein in relation to developing breast cancer has evolved only recently. Recent research in mice showed that low levels of dietary lutein at 0.002 and 0.02% of the diet inhibited mammary tumor incidence, growth and latency [19]. Lutein has been shown to induce apoptosis in transformed but not in normal human mammary cells, and to protect normal cells from apoptosis induced in cell culture [147]. Freudenheim *et al.* [148] have shown that the intake of carotenoid-rich foods, specifically vegetables, as well as lutein and zeaxanthin, is significantly associated with a lower risk of developing premenopausal breast cancer. In a case-control study, increasing serum levels of lutein and zeaxanthin were associated with a reduced breast cancer risk, but the trend was only marginally significant [149]. A decreased risk of cancer was associated with increasing levels of breast adipose tissue lutein and zeaxanthin concentrations in women with breast cancer as compared with women with benign breast biopsies, but the association was not significant [150]. The Nurse’s Health Study [12] showed a weak, but significant, inverse association between lutein and zeaxanthin intake and the risk of developing breast cancer among premenopausal women. The protective effect of lutein and zeaxanthin in relation to breast cancer was strongest among women with a family history of breast cancer. A nested case-control study from the prospective New York University Women’s Health Study [151] indicated an inverse association between plasma lutein, but not zeaxanthin, and risk of breast cancer. However, plasma α- and β-carotene levels were also significantly related to a decrease in risk. Other case-control studies have shown no differences in breast adipose tissue concentrations of lutein and zeaxanthin between women with benign breast tumors and those with breast cancer [152].

### 6.5. Astaxanthin

Because astaxanthin has not typically been identified as a major carotenoid in human serum, information on its epidemiology in human health is lacking. Salmon, the principal dietary source of astaxanthin, is an important component of the traditional diets of Eskimos and certain coastal tribes in North America; these groups have shown an unusually low prevalence of cancer [153,154]. This low cancer incidence has been attributed to the high levels of certain fatty acids in salmon, notably eicosapentaenoic acid [154], yet it is possible that astaxanthin has played a role in cancer chemoprevention among these peoples as well. Regardless, the existing data on the potential for astaxanthin to directly prevent cancer is limited to *in vitro* cell culture studies and *in vivo *studies with rodent models.

We previously investigated the possible preventive effects of astaxanthin and canthaxanthin on *N*-butyl-*N*(4-hydroxybutyl)nitrosamine (OH-BBN)-induced mouse urinary bladder carcinogenesis [155], 4-NQO-induced rat oral carcinogenesis [156] and azoxymethane (AOM)-induced rat colon carcinogenesis [157]. Both of these xanthophylls exhibited inhibitory activity in relation to cancer development in urinary bladder [155], tongue [156] and colorectum [157] through the suppression of cell proliferation. In urinary bladder carcinogenesis, the inhibitory effect of astaxanthin was greater than that of canthaxanthin through the suppression of cell proliferation [155]. A recent study of ours demonstrated the anti-inflammatory ability and anti-carcinogenesis effects of astaxanthin in inflamed colon due to modulation of the expression of several inflammatory cytokines that are involved in inflammation-associated carcinogenesis [158]. Indeed, astaxanthin may aid cyclooxygenase (COX)-2 down-regulation [159]. A recent study using a 1,2-dimethylhydrazine (DMH)-induced colon carcinogenesis model also showed that daily administration of astaxanthin (15 mg/kg body weight) significantly inhibited colon carcinogenesis by modulating nuclear factor kappaB (NF-kB), COX-2, matrix etalloproteinases (MMP) 2/9, extracellular signal-regulated kinase (ERK)-2 and protein kinase B (Akt) [160]. Astaxanthin, canthaxanthin and β-carotene, but not lycopene, are reported to be able to suppressed the development of preneoplastic liver cell lesions induced by AFB_1_ in rats through the deviation of AFB_1_ metabolism towards detoxification pathways [161]. In addition, tetrasodium diphosphate astaxanthin has been reported to completely inhibit methylcholanthrene-induced neoplastic transformation of C3H/10T1/2 cells by upregulation of connexin 43 and gap junctional intercellular communication (GJIC) [162].

### 6.6. Canthaxanthin

Epidemiological data on canthaxanthin in disease prevention is lacking. However, this carotenoid has exhibited potential anticancer properties *in vitro *and in animal models. In earlier studies, canthaxanthin exerted cancer chemopreventive activities in UV-B-induced mouse skin tumorigenesis [163] and chemically-induced gastric [164] and breast carcinogenesis [164,165]. Canthaxanthin can also suppress the proliferation of human colon cancer cells [166], and protect mouse embryo fibroblasts from transformation [167] and mice from mammary and skin tumor development [16,168]. Canthaxanthin has also proved effective in inhibiting both oral and colon carcinogenesis in rats [156,157]. Canthaxanthin and astaxanthin have been found to lower the incidence of urinary bladder cancers induced by OH-BBN, but the inhibitory effects of canthaxanthin were weak when compared to astaxanthin [155]. As was the case with astaxanthin and β-carotene, canthaxanthin suppressed AFB1-induced preneoplastic hepatocellular lesions in rats [161]. Although it is a potent antioxidant, the chemopreventive effects of canthaxanthin may also be related to its ability to up-regulate gene expression, resulting in enhanced gap junctional cell-cell communication [74,169]. The chemopreventive effects of canthaxanthin may also be related to its ability to induce xenobiotic metabolizing enzymes, as has been demonstrated in the liver, lung and kidney of rats [170,171]. The apoptosis-inducing effects of canthaxanthin may also contribute to its cancer chemopreventive effects [172]. Unfortunately, canthaxanthin overuse as a sunless tanning product has led to the appearance of crystalline deposits in the human retina [173]. Although these retinal inclusions are reversible [174] and appear to have no adverse effects [173], their existence has prompted caution regarding the intake of this xanthophyll.

### 6.7. Fucoxanthin

There are several *in vitro* studies that have demonstrated the inhibitory effects of fucoxanthin on human cancer cell lines developed in liver (HepG2) [175], colon (Caco-2, HT-29 and DLD-1) [176] and urinary bladder [177]. The induction of apoptosis [176,177] and the suppression of cyclin D levels [175] have been considered to be the biochemical mechanisms by which fucoxanthin exerts its inhibitory effects on the growth of cancer cells. Since mice actively convert fucoxanthin into keto-carotenoids by oxidizing the secondary hydroxyl groups and accumulating them in tissues [178], it may be possible that keto-carotenoids are active chemicals responsible for the effects of fucoxanthin.

In a preclinical study, fucoxanthin was found to significantly inhibit DMH-induced mouse colon carcinogenesis [179]. Fucoxanthin has been proven to suppress spontaneous liver tumorigenesis in C3H/He male mice and showed antitumor-promoting activity in a two-stage carcinogenesis experiment involving the skin of ICR mice, initiated with 7,12-dimethylbenz[*a*]anthracene and promoted with 12-*O*-teradecanoylphorbol-13-acetate and mezerein [180]. In addition, fucoxanthin has been reported to inhibit duodenal carcinogenesis induced by *N*-ethyl-*N*′-nitro-*N*-nitrosoguanidine in mice [181]. 

Although the antitumor effects of fucoxanthin are known, the precise mechanism of action has yet to be elucidated [182]. The anticancer activity of fucoxanthin has been shown to be partly based on its regulative effect on biomolecules related to the cell cycle and apoptosis [183,184] and those associated with antioxidant activity through its pro-oxidant action [185]. In addition, fucoxanthin has been found to be able to selectively inhibit mammalian DNA polymerase activities, especially replicative DNA polymerases (*i.e.*, pol α, δ and ε), and thus has anti-neoplastic activity [186]. Further investigations using animal models are needed to assess the details of the molecular mechanisms involved in fucoxanthin’s activity against different types of cancer cells. 

## 7. Mechanisms of Cancer Chemoprevention by Carotenoids

**Figure 2:**
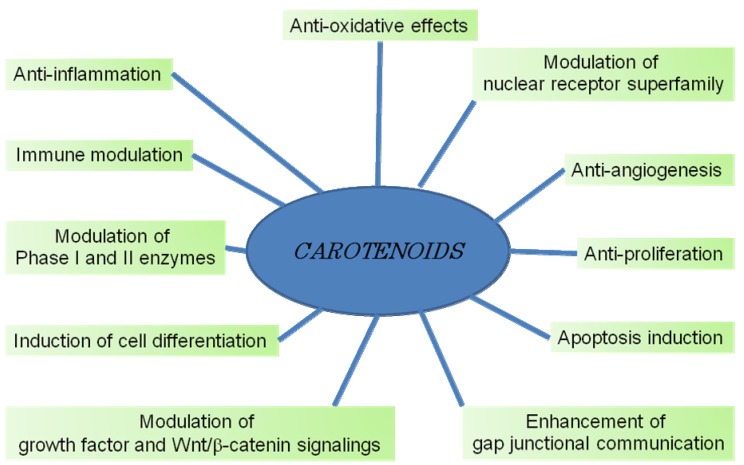
Proposed mechanisms by which certain carotenoids suppress carcinogenesis.

The mechanisms underlying the anticancer and/or cancer chemopreventive activities of carotenoids may involve changes in pathways leading to cell growth or cell death. These include immune modulation, hormone and growth factor signaling, regulatory mechanisms of cell cycle progression, cell differentiation and apoptosis. Examples of carotenoid effects on some of these pathways are listed below, with the emphasis being placed on the changes in protein expression associated with these effects. The main question is, by what mechanism do carotenoids affect so many and diverse cellular pathways as described above? The changes in the levels of many proteins suggest that the initial effect involves modulation of transcription. As described below, such modulation can occur at the level of ligand-activated nuclear receptors or other transcription factors. As illustrated in Figure 2, carotenoids have multiple targets that contribute to their efficacy as chemoprevention agents.

### 7.1. Gap Junctional Intercellular Communication

One of the earliest discoveries related to carotenoids and modulation of protein level was made by Bertram’s group. They found that carotenoids increase gap junctional intercellular communication (GJIC) and induce the synthesis of connexin43, a component of the gap junction structure [74,187]. This effect was independent of provitamin-A and the antioxidant properties of the carotenoids. Loss of GJIC may be important for malignant transformation, and its restoration may reverse the malignant process.

### 7.2. Growth Factor Signaling

Growth factors, either in the blood or as part of autocrine or paracrine loops, are important for cancer cell growth. Recently, insulin growth factor (IGF)-1 has been implicated as a major cancer risk factor [188,189] and a target of potential for dietary intervention strategies for cancer prevention [189]. It has been reported that high blood levels of IGF-1, existing years before the detection of malignancy, can predict an increase in risk for prostate [190], breast [191], colorectal [192] and lung [193] cancers. A recent study of ours indicated that *db/db*-*Apc^Min/+^* with increased expression of IGF-1, IGF-1R and IGF-2 in the intestine was associated with an increased incidence of spontaneous intestinal neoplasms [194]. Accordingly, two possible strategies might be used to reduce IGF-related cancer risk, namely a reduction in IGF-1 blood levels and interference with IGF-1 activity in the cancer cell. Preliminary results of our studies on the former strategy suggest that tomato phytonutrients lower IGF-1 blood levels. In addition, lycopene inhibits the mitogenic action of IGF-1 in human cancer cells. In mammary cancer cells, lycopene treatment markedly reduced IGF-1 stimulation of both tyrosine phosphorylation of insulin receptor substrate-1 and the DNA binding capacity of the activator 1 (AP-1) transcription factor [195]. These effects were not associated with changes in the number or affinity of IGF-1 receptors, but rather with an increase in membrane-associated IGF binding proteins (IGFBPs). This finding can explain the suppression of IGF-1-signaling by lycopene based on the finding that membrane-associated IGFBP-3 inhibits IGF-1 receptor signaling in an IGF-dependent manner [196].

### 7.3. Cell Cycle Progression

Growth factors have a major effect in promoting cell cycle progression, primarily during the G1 phase. Lycopene treatment of MCF-7 mammary cancer cells has been shown to slow down IGF-1-stimulated cell cycle progression [195], which was not accompanied by either apoptotic or necrotic cell death. Lycopene-induced delay in progression through the G1 and S phases has also been observed in other human cancer cell lines (leukemia and cancers of endometrium, lung and prostate) [197]. Similar effects of another carotenoid, α-carotene, were reported in human neuroblastoma cells (GOTO) [198]. Likewise, β-carotene was found to induce a cell-cycle delay in the G1 phase in normal human fibroblasts [199]. Fucoxanthin is reported to alter cell cycle progression [182,184,200]. In addition, metabolites of lycopene, apo-10'-lycopenoic acid [118] and apo-12'-lycopenal [201] can induce cell cycle arrest in cancer cells. Cancer cells arrested by serum deprivation in the presence of lycopene are incapable of returning to the cell cycle after serum re-addition [202]. This inhibition correlated with a reduction in cyclin D1 protein levels that resulted in inhibition of both Cdk4 and Cdk2 kinase activity and in hypophosphorylation of pRb. 

### 7.4. Differentiation-Related Proteins

Induction of malignant clonogenic cells to differentiate into mature cells with distinct functions similar to those of nonmalignant cells has been proposed as an alternative to cytotoxic chemotherapy, and may be useful for chronic chemoprevention. Differentiation therapy has been quite effective in treating acute promyelocytic leukemia and is currently being investigated for the treatment of solid tumors. Differentiation inducers that are presently under laboratory and clinical investigation include vitamin D and its analogs, retinoids, polyamine inhibitors and others. We have shown that lycopene alone induces differentiation of HL-60 promyelocytic leukemia cells [197]. A similar effect has also been described for other carotenoids such as β-carotene, lutein and the saffron carotenoids [197,203,204]. The differentiation effect of lycopene was associated with elevated expression of several differentiation-related proteins such as cell surface antigen (CD14) and oxygen burst oxidase (as measured by phorbol ester-stimulated reduction of nitroblue tetrazolium) [197]. The mechanism of the differentiating activity of lycopene and its ability to synergize with 1,25(OH)_2_D_3_ in this effect [197] is largely unclear. However, the differentiation-enhancing effect of another phytonutrient, carnosic acid from rosemary, is associated with the induction of multiple differentiation-related proteins such as Cdk inhibitor, p21Cip1, early growth response gene (EGF)-1 and Cdk5 and its activator protein, p35Nck5a [205,206]. Most importantly, carnosic acid and its combinations with 1,25(OH)_2_D_3_ and retinoic acid transcriptionally activated the expression of nuclear hormone receptors such as vitamin D_3_ receptor (VDR), retinoic acid receptor (RAR) α, and retinoid X receptor (RXR) α [205,206]. This may represent a molecular basis for synergy between phytonutrients and differentiation inducers. The possibility that lycopene, as well as other carotenoids and/or their derivatives, may affect nuclear signaling pathways is an attractive suggestion, but requires experimental proof.

### 7.5. RAR

The structural similarity between lycopene and β-carotene suggests that lycopene or some of its oxidized derivatives may activate retinoid-like receptors. Acyclo-retinoic acid, a hypothetical oxidation product of lycopene, is the open chain analog of retinoic acid [207] and was found to be able to transactivate RARα, but the growth-inhibitory effect of lycopene was not mediated directly via this classical retinoid receptor [208]. In addition, acyclo-retinoic acid has been reported not to have a role in gap junctional communication [207]. Muto *et al.* [209] synthesized acyclo-retinoic acid and tested its biological activity as part of a series of acyclic retinoids, but did not observe transactivation by this compound in the RAR or RXR reporter gene systems [210]. However, they did find that other acyclic retinoids, lacking one or two double bonds (geranyl geranoic acid and 4,5-didehydrogeranylgeranoic acid), caused transactivation of the reporter gene comparable to that achieved by retinoic acid. It is interesting to note that these acyclic retinoids may be potential derivatives of phytoene and phytofluene carotenoids present in tomatoes. These studies suggest that carotenoids, their oxidized derivatives, and other phytonutrients interact with a network of transcription factors that are activated by different ligands at low affinity and specificity. The activation of several transcription factor systems by different compounds may lead to the synergistic inhibition of cell growth. In addition to the retinoid receptors, other candidate transcription systems that may participate in this network are the peroxisome proliferator-activated receptors (PPARs) [211,212,213,214], ARE [215,216], AP-1 [217], the xenobiotic receptors [218] and yet unidentified orphan receptors.

Recent elucidation of the pathways that are activated by retinoids will help to exploit the beneficial aspects of this class of compounds for cancer therapy and prevention [219,220]. Retinoids and carotenoids are important dietary factors which regulate cellular differentiation and growth, so that they are thought to be particularly effective at preventing the development of certain tumors. They play this role as ligands of the nuclear retinoic acid receptors, RAR and RXR [220]. These ligand-activated nuclear receptors induce the transcription of target genes by binding to retinoic acid-responsive elements in the promoter regions. Among these target genes, the RARβ gene is of great interest, being able to encode a potential tumor suppressor. It should be emphasized that most breast carcinomas and breast cancer cell lines show loss or down-regulation of RARβ receptor expression, whereas RARα and γ, as well as RXRs, appear to be variably expressed in both normal and tumor cells [220]. Expression of RARβ could be modulated by chemopreventive intervention [221,222] and may therefore serve as an intermediate biomarker in chemoprevention trials for some cancers [223]. Provitamin A carotenoids, such as β-carotene and its excentric cleavage metabolites, can serve as direct precursors for (all-*trans*)-retinoic acid and (9-*cis*)-retinoic acid which are ligands for RAR and RXR, respectively. β-Carotene and its oxidative metabolite, apo-14'-carotenoic acid, are reported to reverse the down-regulation of RARβ by smoke-borne carcinogens in normal bronchial epithelial cells [224]. In addition, the transactivation of the RARβ promoter by β-apo-14'-carotenoic acid appears to occur via its metabolism to all-*trans*-retinoic acid [224]. Therefore, the molecular mode of the action of β-carotene might be mediated by retinoic acid through transcriptional activation of a series of genes with distinct anti-proliferative or pro-apoptotic activity, which allows for the elimination of neoplastic and preneoplastic cells with irreparable alterations.

### 7.6. PPAR

These nuclear receptors have a key role in the differentiation of adipocytes, but recently their role in cancer cell growth inhibition and differentiation has also been demonstrated. PPARγ is expressed at significant levels in a variety of human primary and metastatic carcinomas [214,225,226,227]. Human colorectal cancer was found to be associated with loss-of-function mutations in PPARγ [228]. Ligand activation of PPARγ was reported in cultured breast cancer cells [213]. Human prostate cancer cells have been shown to express PPARγ at prominent levels, while its expression in normal prostate tissues was very low [212,213]. Activation of this receptor with specific ligands such as troglitazone exerts an inhibitory effect on the growth of prostate cancer cells, and favorable changes in PSA dynamics in prostate cancer patients [213]. The presence of PPARγ receptors in various cancer cells, their activation by fatty acids, prostaglandins and related hydrophobic agents in the μM range makes this liganded transcription factor an interesting target for carotenoid derivatives. We have previous demonstrated that fucoxanthin can induce apoptosis and enhance the antiproliferative effects of the PPARγ ligand, troglitazone, and inhibit the growth of human colon cancer cells [176].

Recently, Simone *et al*. [229] reported new molecular mechanisms by which lycopene regulates cigarette smoke-driven inflammation in human macrophages, THP-1. They have shown that lycopene inhibits the production of the pro-inflammatory cytokine interleukin (IL)-8 induced by cigarette smoke. More recently, Yang *et al*. [230] demonstrated that the anti-proliferative effect of lycopene on human prostate cancer cells (LNCaP) involves the activation of the PPARγ-LXRα-ATP-binding cassette transporter 1 (ABCA1) pathway.

### 7.7. Xenobiotic and other Orphan Nuclear Receptors

Orphan receptors include gene products that are structurally related to nuclear hormone receptors, but lack known physiological ligands. Thus, like all the recognized nuclear receptors they should have multiple regulatory roles, some of which may be related to diet-derived compounds. Mammals encounter numerous xenobiotics which are metabolized and eliminated mainly by cytochrome P450 (CYP) enzymes [218]. CYP enzymes are induced by various xenobiotic substrates, including phytonutrients, through the response element of several orphan nuclear receptors such as the steroid and xenobiotic receptor/pregnane X receptor (SXR/PXR), and the constitutive androstane receptor (CAR) [218,231]. St. John’s wort, the herbal remedy used widely for the treatment of depression, illustrates the possible role of phytonutrients in this system. It has recently been found that its active compound, hyperforin, is a potent ligand for PXR that promotes the expression of CYP 3A4 [232].

### 7.8. Antioxidant Response Element

Induction of phase 2 enzymes that neutralize reactive electrophiles and act as indirect antioxidants appears to be an effective means for achieving protection against a variety of carcinogens in animals and man. Transcriptional control of the expression of these enzymes is mediated, at least in part, through the antioxidant response element (ARE) found in the regulatory regions of their genes. The transcription factor Nrf2, which binds to the ARE, appears to be essential for the induction of prototypical phase 2 enzymes such as glutathione *S*-transferases (GSTs), NAD(P)H:quinone oxidoreductase (NQO1) [233] and thioredoxin [234]. The constitutive hepatic and gastric activities of GST and NQO1 were reduced by 50–80% in Nrf2-deficient mice as compared with wild-type mice [65]. Under basal conditions, Nrf1 and Nrf2 are located in the cytoplasm and are bound to the inhibitory protein, Keap1. Upon challenge with inducing agents, they are released from Keap1 and translocate to the nucleus [235,236]. Within the nucleus, these basic region leucine zipper transcription factors are recruited to the ARE as heterodimers with either small Maf proteins, FosB, c-Jun or JunD. Several studies have shown that dietary antioxidants such as terpenoids [237], phenolic flavonoids including green tea polyphenols and epigallocatechin-3-gallate [238,239] and isothiocyanates, may work as anticancer agents by activating this transcription system. By way of illustration, an isothiocyanate compound from Japanese horseradish extract has been demonstrated to induce both nuclear localization of Nrf2, which binds to the ARE, and expression of phase 2 enzyme genes. These effects were completely abrogated in Nrf2-deficient mice [240].

### 7.9. AP-1 Transcriptional Complex

The activation of the AP-1 transcriptional complex is a middle-term event (1–2 h) in the mitogenic signaling pathway of IGF-1 and other growth factors [241]. The AP-1 complex consists of protein from the Jun (c-Jun, JunB and JunD) and Fos (c-Fos, FosB, Fra-1 and Fra-2) families, which associate as homo- (Jun/Jun) or heterodimers (Jun/Fos). These proteins are often induced by mitogenic stimuli and tumor-promoting agents. They bind to the AP-1 site, known also as the TPA response element (TRE), on the promoter of many genes that are related to cell proliferation such as cyclin-D [242]. Interestingly, some of these proteins participate in the ARE transcription complex as well. This transcriptional system is modulated by carotenoids. It is possible that lycopene and retinoic acid reduce growth factor-induced stimulation of AP-1 transcriptional activity by altering the composition of AP-1 complexes that bind to DNA [39,243]. Wang *et al.*[217] reported that the expression of c-Jun and c-Fos genes in the lungs of ferrets, supplemented with high-dose β-carotene and exposed to tobacco smoke, was elevated 3- to 4-fold. In addition, they observed a strong proliferative response in lung tissue and squamous metaplasia, as well as an increase in the level of a cell proliferation marker, proliferating cell nuclear antigen. In β-carotene-supplemented animals, this increase was enhanced further by tobacco smoke. Their report offers a possible explanation for the enhancing effect of β-carotene supplementation on lung carcinogenesis in smokers, as has been reported in large intervention studies [13,15].

### 7.10. Wnt/β-Catenin Pathway

The Wnt/β-catenin pathway has been demonstrated to modulate cell proliferation, migration, apoptosis, differentiation and stem cell self-renewal [244]. It has been shown that Wnt/β-catenin signaling is implicated in the maintenance of stem cells in a variety of cancers, including colorectal cancer [245]. The link between Wnt/β-catenin and the PI3K/Akt pathway has been established by several studies. Activated Akt was shown to be able to phosphorylate Ser9 on glycogen synthase kinase 3β (GSK3β), which may decrease the activity of GSK3β, thereby stabilizing β-catenin [246]. Furthermore, the PI3K/Akt pathway is important in regulating the mammary stem/progenitor cells by promoting β-catenin downstream events through the phosphorylation of GSK3β [247]. In colon cancer cells, lycopene suppressed Akt activation and nonphosphorylated β-catenin protein levels, and augmented the phosphorylated form of β-catenin, which were associated with reduced protein expression of cyclin D1 [248]. Hence, lycopene may inhibit Wnt/β-catenin signaling via the connection along the Akt/GSK3β/β-catenin [249]. Further studies on cancer stem cells in response to lycopene would perhaps provide promising new data.

### 7.11. Inflammatory Cytokines

Cancer frequently develops in inflamed tissues, suggesting that the inflammatory condition is closely related to carcinogenesis [250,251]. Examples of this relationship are: chronic hepatitis (HBV and HCV infection) and liver cancer; Barrett dysplasia and esophageal cancer; chronic gastritis (*H. pylori* infection) and gastric cancer; and inflammatory bowel disease and colorectal cancer [250]. The common denominator of all these conditions is that chronic inflammation leads to an increased incidence of cancer [250]. Thus, suppression of inflammatory cytokine expression leads to inhibition of carcinogenesis. These inflammatory cytokines include IL-1β, IL-6 and tumor necrosis factor (TNF)- α. Cytokine expression is mainly regulated by NF-κB. A recent study of ours demonstrated that astaxanthin suppressed the expression of these inflammatory cytokines and NF-κB, and inhibited inflammation-associated colon carcinogenesis in mice [158]. In addition, lycopene is reported to inhibit pancreatitis [252]. Chronic pancreatitis and hereditary pancreatitis are believed to increase the risk of pancreatic cancer [253,254].

## 8. Conclusions

Beneficial effects of carotenoid-rich vegetables and fruits in relation to cancer risk have been found in many epidemiological studies. However, the metabolism and molecular biological properties of carotenoids remain to be determined through further research. Provitamin A carotenoids (α-carotene, β-carotene and β-cryptoxanthin) combined with other antioxidants (ascorbic acid, α-tocopherol and lycopene) limit the oxidative cleavage products of carotenoids, formed in large quantities in the highly oxidative conditions of the smoke-exposed lung and enhance retinoid signaling, by blocking the activation of MAPK. In considering the efficacy and complex biological functions of carotenoids in the prevention of human lung cancer [255], it seems that these provitamin A carotenoids and antioxidants used in combination could be employed as a chemopreventive strategy against certain human cancers. However, there appear to be detrimental interactions between β-carotene, cigarette smoke and alcohol. In addition, the molecular mechanisms that underlie these interactions need to be understood before β-carotene can be further pursued for the prevention of carcinogenesis in man. As we await a better scientific understanding of carotenoid metabolism and the mechanisms of action, a prudent strategy to reduce the risk of cancer incidence and mortality would include increased consumption of vegetables and fruits as a part of a healthy, balanced diet. This would include eating between five to nine servings of fruits and vegetables every day. There is currently no evidence of any dangers associated with high levels of dietary β-carotene from natural food sources, aside from the occasional appearance of carotenodermia, an accumulation of β-carotene in the skin that gives it a yellow or orange tint. At present, supplemental doses of β-carotene taken to meet vitamin A needs beyond the recommended dietary intake dose are not advisable for the general population. Smokers and alcohol drinkers are especially encouraged to avoid high doses of supplemental β-carotene.
